# Analysis of Return-to-Zero Error after the First Load of Load Cell

**DOI:** 10.3390/s23218712

**Published:** 2023-10-25

**Authors:** Shudong Zhuang, Wen Yang, Xianming Cheng, Jenny Sama Kevin, Chang Liu, Guangjie Zhang, Wenbin Zhu, Chengdong Tian

**Affiliations:** 1 College of Mechanical and Electrical Engineering, Hohai University, Changzhou 213022, China; zhsd1970@126.com (S.Z.); cxm@hhu.edu.cn (X.C.); zhanggj@hhu.edu.cn (G.Z.); w.b.zhu@hhu.edu.cn (W.Z.); tiancd@hhu.edu.cn (C.T.); 2 College of Materials Science and Engineering, Hohai University, Changzhou 213022, China; 3 Department of Physics, California San Diego University, San Diego, CA 92127, USA; chl143@ucsd.edu

**Keywords:** zero-point error, hysteresis, Pearson correlation coefficient, data fitting algorithm

## Abstract

The return-to-zero error of the resistance strain load cell is most obvious in the first zero-return process during loading and unloading. To improve the accuracy of the load cell, it is necessary to figure out the cause of the error. The influence of the temperature, material, and weld cup were analyzed in this paper. It was concluded that the hysteresis is the main factor affecting the return-to-zero error after the first load. The relationship between hysteresis and zero-return error after first load was obtained by a data fitting algorithm. A method to improve the return-to-zero error after the first load was proposed.

## 1. Introduction

Resistance strain load cell is a kind of mechanical measurement sensor which is wildly used in various field such as industrial robotics [1,2], agriculture [3,4,5], medicine [6,7,8,9], sports industry [10,11], and many high-precision weighing applications [12,13,14,15,16,17]. It is a sensitive component that converts the deformation of the strain gage under force into an electrical signal [18]. Resistance strain gauges are tightly attached to the surface of the metal elastomer with a special adhesive. When the elastomer is deformed by the force, the strain gauges are deformed as well, resulting in a change in the resistance of the strain gauges. Through the Wheatstone bridge, the external force is changed into an electrical signal. Due to the inherent properties such as hysteresis and creep, it is extremely challenging to manufacture sensors with high sensitivity, wide sensing range, high repeatability, robustness and continuous production [19].

In practical use and research, resistance strain sensors are subject to return-to-zero errors after first load. This phenomenon is caused by the hysteresis of the sensor system after study. Hysteresis is a complex phenomenon [20] with multiple causes. Mei H et al. proposed that hysteresis may be caused by material properties [21], and Berry et al. proposed that it may also be caused by mechanisms [22,23] or other factors [24]. In order to solve the problems caused by sensor hysteresis, researchers have proposed a variety of hysteresis compensation techniques. These techniques could be categorized into two main groups: model-based compensation methods and data-based compensation methods. Under the model-based compensation methods, a mathematical model is used to describe the hysteresis characteristics of the sensor and the compensation is performed based on the model. Common models include the Prandtl–Ishlinskii model [25], the Inverse Preisach model [26], and so on. Data-based compensation methods, on the other hand, do not require a detailed modeling of the sensor, while they compensate the error based on actual measurement data instead [27]. These methods typically utilize statistical properties of the hysteresis curve or signal processing techniques to estimate the hysteresis and compensate for it.

In OIML R76-1 [28] and OIML R60 [29], it is stated that load cells need to be preloaded before use. However, in practice, few users preload the scale before use, which leads to return-to-zero error. This is reflected in the fact that all zeros after the first zero point are a non-zero number, which leads to a situation in which the indicated value of the scale is different from the weight of the object itself. With the development of science and technology, people have more requirements for the accuracy of the weighing sensor in some specific occasions. Therefore, reducing the return-to-zero error after the first loading has become an urgent problem.

Reference [30] focuses on the relationship between the zero error and creep of load cells. However, the effect of hysteresis was not considered. In reference [31], a method for compensating the return-to-zero error after the first load of a load cell was studied. But the underlying cause—hysteresis—was not investigated. In this paper, through theoretical analysis and a large number of experiments, the relationship between the return-to-zero error after the first load of the load cell and the main influencing factor—hysteresis—is listed, which helps the investigation of the zero-point error of the load cell.

The characteristics and principles of the return-to-zero error of load cell are analyzed in Section 2, and the steps of the experiment are listed. The experimental results are listed in Section 3, where the linear correlation of the experimental results is analyzed, and the relationship between the hysteresis and the first time zero-return error is calculated. Section 4 verifies the correctness of the equation relating hysteresis to the return-to-zero error after the first load. The factors that produce the return-to-zero error after the first loading are summarized and the outlook is presented in Section 5.

## 2. Characterization and Principle Analysis of Return-to-Zero Error

### 2.1. Zero-Return Characteristics of Load Cells

**Figure 1:**
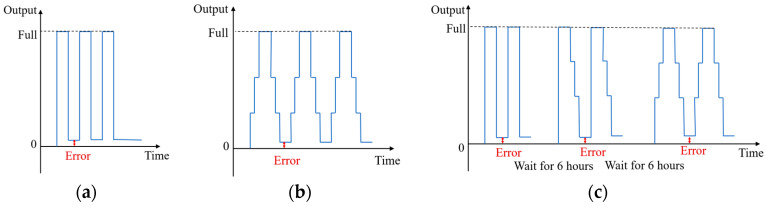
Characteristics of return to zero for multiple loading methods: (**a**) Directly to full loading; (**b**) Stages to full loading. (**c**) Multiple methods of full loading.

The return-to-zero error after the first loading occurs almost exclusively at the first time, after which the return-to-zero tends to stabilize. There is basically no difference between the values of the zero points. With the sensor resting for 6 h, the zeroing error reoccurs after the first loading, as shown in Figure 1.

The return-to-zero error after the first load of the sensor is always present, regardless of how loading and unloading is performed. During the subsequent loads, the return-to-zero error is minimal or almost non-existent. However, the error appears again after sitting for 6 h.

### 2.2. Factors Generating Return-to-Zero Error after First Loading

**Figure 2:**
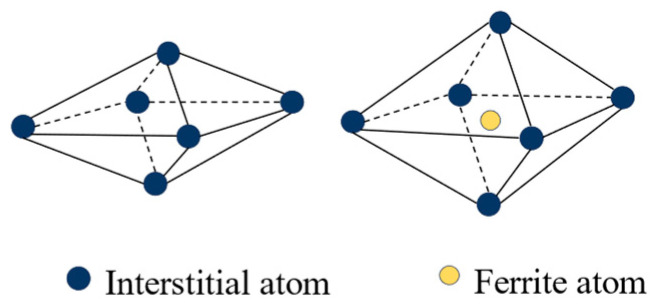
Illustration of the interstitial atom in octahedron of a bcc crystal.

**Figure 3:**
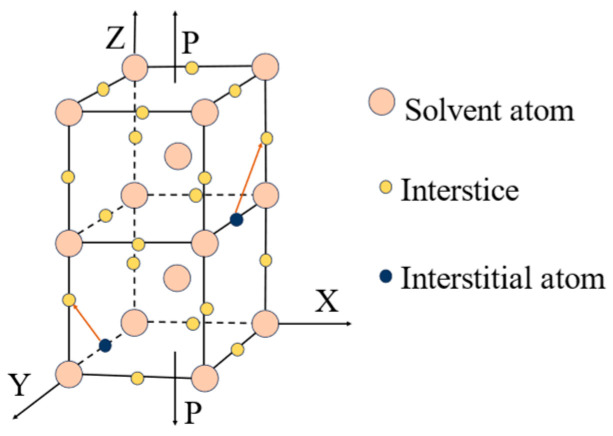
Stress-induced reorientation of interstitial atoms in bcc crystals.

At the microscopic level, metallic materials contain a large number of point defects, line defects and surface defects that contribute to the phenomenon of return-to-zero error after the first load. From an energetic point of view, the interstitial atoms cause distortions in the crystal, and these defects are unstable. When there is no applied stress, the interstitial atoms are distributed between the crystals without any specific order, and the degree of distortion is uniform in all directions within the material. However, when the metal is subjected to an external force, in an effort to minimize distortion energy, the interstitial atoms follow a certain diffusion pattern and exhibit a preferential distribution. This phenomenon is known as stress-sensitive ordering [32]. With the stress- sensitive ordering phenomenon having occurred, a large number of atoms are diffused and rearranged. If the pressure is now applied again, the atoms will not diffuse further but will remain in the position where they initially diffused. This is why the return-to-zero error occurs only during the first time. Over time, the interstitial atoms slowly return to their original positions. When pressure is applied to the metal at this time, the interstitial atoms will diffuse again following a certain pattern. The return-to-zero error after loading reappears. The diffusion of the atoms is illustrated in Figure 2 and Figure 3.

**Figure 4:**
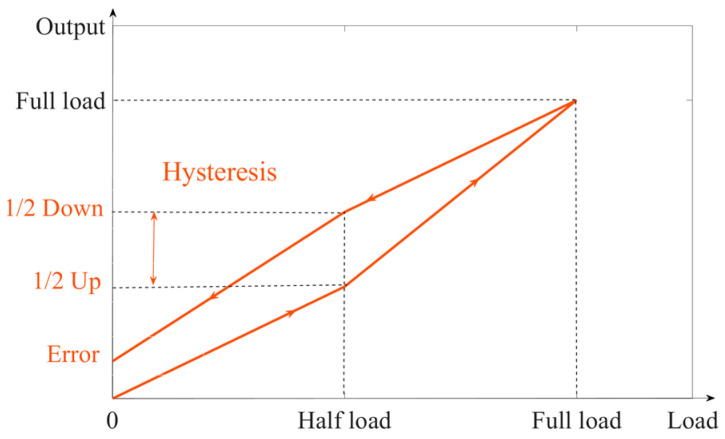
Sensor return-to-zero curve.

From a macroscopic perspective, this is caused by the hysteresis of the sensor system. Stress-sensing ordering and hysteresis are inherent characteristics of the material itself. Hysteresis refers to the phenomenon in which the properties or characteristics of a material do not fully return to their initial state during the loading and unloading process. When external stresses are applied to a material, its atomic, molecular, or lattice structure changes, resulting in the generation of strain. These structural changes do not occur instantaneously, but exhibit some delay after the application of stress. The hysteresis of a sensor manifests as the difference between the sensor’s output readings when the same load is applied during loading and unloading. This deviation is not completely linear due to the hysteresis effects. Typically, the most significant hysteresis occurs at half load. The loaded and unloaded zero curve of the sensor is shown in Figure 4.

It can be seen that hysteresis is the difference between the output when loaded to half load and when unloaded to half load. There is a relationship between the return-to-zero error after the first load and the hysteresis. On the unload curve, if the hysteresis is larger and the slope of the curve is constant, the return-to-zero error after the first load is larger. If the hysteresis is smaller and the slope of the curve is constant, the return-to-zero error after the first load is smaller.

### 2.3. Design of the Experiment

#### 2.3.1. Temperature Experiments

References [33,34] indicate that the effect of temperature on hysteresis is almost negligible. Therefore, the effect of temperature on the return-to-zero error after the first load is not significant without considering the zero drift.

In order to verify the effect of temperature on the zero return after the first loading, experiments were performed using a constant temperature chamber with the temperature range of −10 °C to 40 °C. The experiments were designed as follows:

(1) Set the temperature of the chamber to 20 °C. The chamber temperature was stabilized and maintained for 24 h. The sensor was loaded and unloaded at full scale and each zero point was recorded.
(2) The temperature of the chamber was increased from 20 °C to 40 °C. The chamber temperature was stabilized and maintained for 24 h. The sensor was loaded and unloaded at full scale and each zero point was recorded.
(3) The temperature of the chamber was decreased from 40 °C to −10 °C. The chamber temperature was stabilized and maintained for 24 h. The sensor was loaded and unloaded at full scale and each zero point was recorded.
(4) The temperature of the chamber was increased from −10 °C to 5 °C. The chamber temperature was stabilized and maintained for 24 h. The sensor was loaded and unloaded at full scale and each zero point was recorded.
(5) The temperature of the chamber was increased from 5 °C to 20 °C. The chamber temperature was stabilized and maintained for 24 h. the sensor was loaded and unloaded at full scale and each zero point was recorded.

Set the first zero point before the experiment as the zero scale. The change in the zero point after loading and unloading at each temperature was compared separately to observe the change in the zero point after the first loading of the sensor when warming up or cooling down.

#### 2.3.2. Experiments with and without Weld Cup

The weld cup is a component commonly used for load cell sealing, and its material properties are usually identical to those of the load cell. Especially in the fabrication of steel sensors, weld cup seals have become more common, and it has become a more mature sealing method [35].

**Figure 5:**
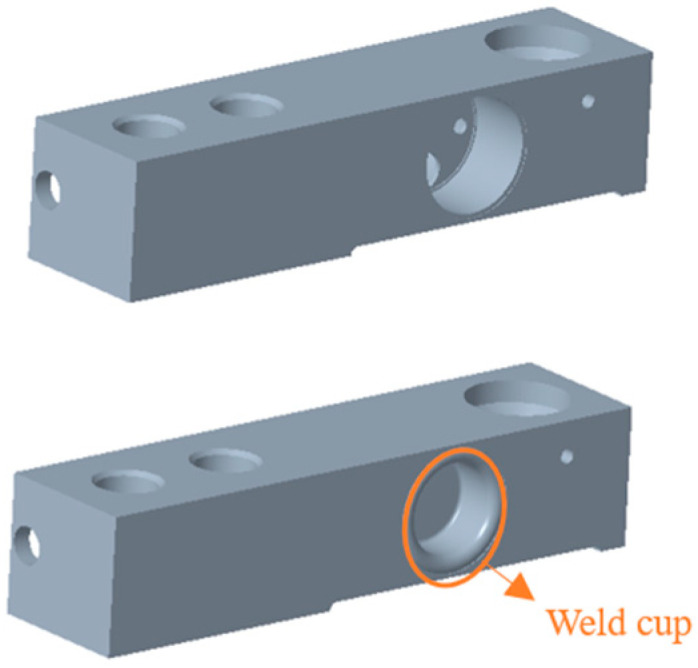
Sensor without weld cup (**top**) and sensor with weld cup (**bottom**).

The welding position of the weld cup is shown in Figure 5.

To study the effect of the weld cup on the performance of the sensor, the model was modeled using Creo Parametric and then imported into the finite element analysis software ANSYS. The material settings of the elastomer are shown in Table 1 (weld cup materials are usually the same as those of elastomer).

**Figure 6:**
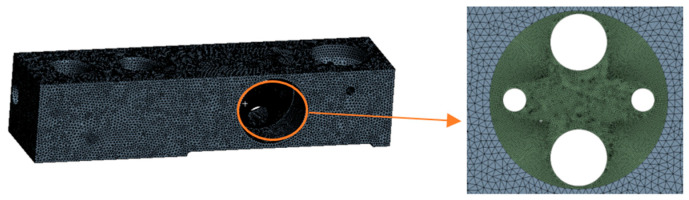
Result of finite element analysis meshing.

After importing the model and setting the material properties, the middle strain region was cut into two geometries and then meshed. After considering the accuracy and computational complexity, the elastomer was meshed by the Gnenrate Mesh method first. Then, the strain zone was meshed by the Sweep method. Because of the stress concentration in the strain region, the middle strain region was encrypted to improve the calculation accuracy, and the final meshing results are shown in Figure 6.

**Figure 7:**
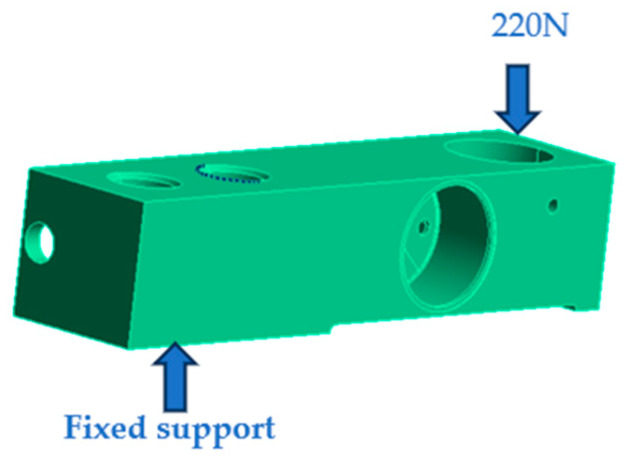
Setting the sensor constraints.

We set the constraints as shown in Figure 7.

Finite element analysis was carried out for the sensors with and without weld cups, respectively. After the results were obtained, strain extraction was carried out for the strain region to compare the strains.

**Figure 8:**
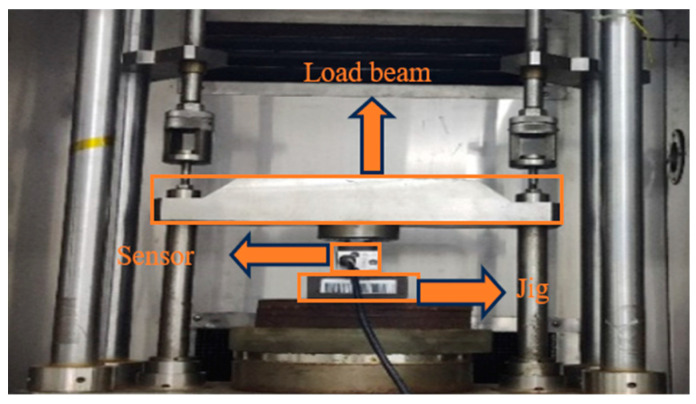
Force machine loading diagram.

Finally, sensors with and without weld cups were experimented with on a static gravity machine with a unit weight of 100 kg. The main components of the force machine included a loading beam for applying the weight. The fixture for fixing the load cell is shown in Figure 8.

#### 2.3.3. Experiments with Different Elastomer Materials

Common load cell according to the elastomer material points have steel sensors and aluminum sensors. Steel sensors and aluminum sensors have different metal properties. The strength of steel is greater than that of aluminum; therefore, steel sensors can measure heavier objects. The range is also larger, but the accuracy is lower than that of aluminum sensors. Aluminum sensors can measure lighter objects. The range is smaller, but relative to the steel sensor, the accuracy is higher. 

**Figure 9:**
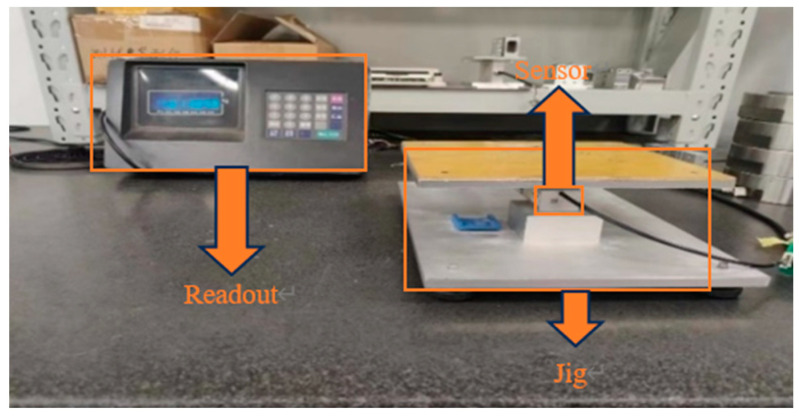
Aluminum sensor mounting and positioning diagram.

Experiments were carried out with five steel sensors with a range of 220 kg and five aluminum sensors with a range of 10 kg. Experiments for the steel sensors are shown in Figure 8. Experiments for the aluminum sensors as shown in Figure 9. 

The experimental equipment for the aluminum sensors consisted mainly of a fixture for fixing the aluminum sensors and a reading instrument, which was fitted with an experimental plane on which weights can be placed.

After all the sensors were fully loaded and then unloaded to derive the first return-to-zero error, all the first return-to-zero error data were compared. The hysteresis of each sensor are listed and the data are analyzed.

## 3. Results and Discussion

### 3.1. Temperature

The experimental results of the return-to-zero error after the first load of all sensors are shown in Table 2.

The change in zero point after loading and unloading at each temperature was taken out for comparison, as shown in Table 3.

**Figure 10:**
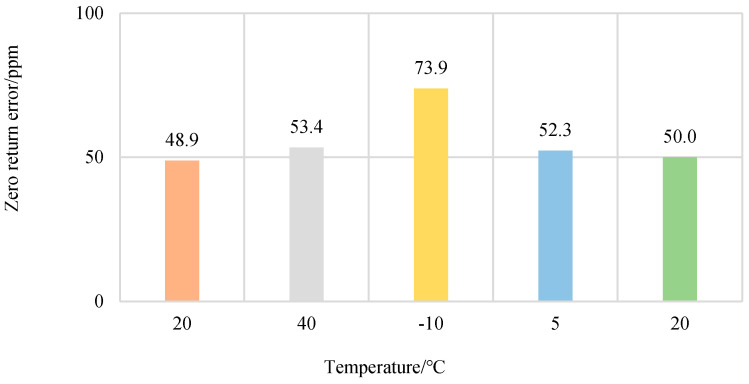
Return-to-zero error after the first load at each temperature.

The data from the two sensors with different ranges could not be compared directly, so the data were converted to a “ppm” plot and then compared [36]. Designation ”1 ppm” means one part per million, and “ppm” is commonly used as an indicator for precision instruments; thus, the zero change is converted to a “ppm” graph first, as can be seen in Figure 10.

The experimental results show that the change in temperature does not have a significant effect on the return-to-zero error after the first load. This is due to the fact that the temperature has very little effect on the hysteresis, which leads to this result.

### 3.2. Weld Cup

**Figure 11:**
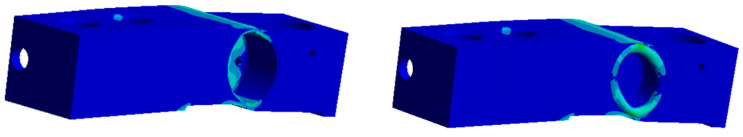
Sensors strain diagrams with (**right**) and without (**left**) weld cup.

The strain obtained by the experiment in Section 2.3.2 with and without the welding cup sensor is shown in Figure 11.

Further examining the analysis, since the sensor is a shear sensor, its strain is maximum at the oblique 45 degrees in the strain region. The strain gauges are also placed at this location; therefore, a path with the same start, length, and end point is added at the oblique 45-degree angle of the sensor with and without the weld cup, respectively.

**Figure 12:**
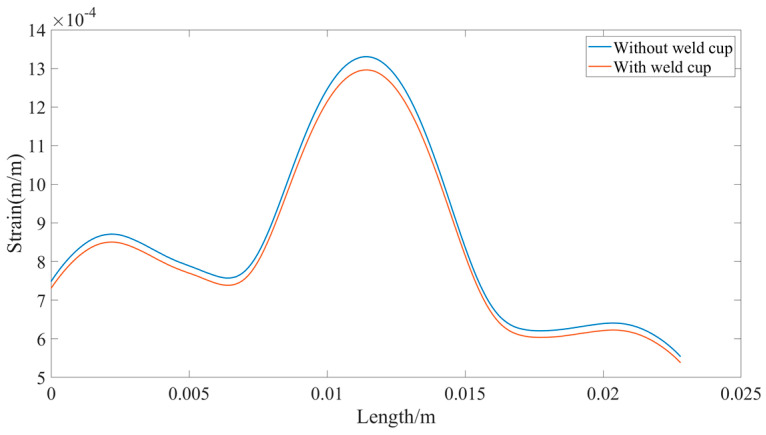
Strain in the strain zone for sensor with and without weld cup.

The equivalent strain on this path is extracted for the two sensors with and without weld cup, and the strain is analyzed numerically with the starting point as the zero-scale point, as shown in Figure 12.

It can be seen that the sensor without the weld cup has a larger strain in the strain region compared to the sensor with the weld cup. This means that the accuracy of the sensor is affected by the weld cup. Further experiments are conducted to demonstrate whether the solder cups have an effect on the return-to-zero error after the first load of the load cell.

Therefore, the two sensors are first preloaded to eliminate residual mounting stresses. After 24 h, without preloading, the zero point $Z_{1}$ is taken before the experiment, and *Z*_1_ is defined as zero. Full loading and unloading are performed, and five zero points, $Z_{1}$, $Z_{2}$, $Z_{3}$, $Z_{4}$, $Z_{4},$ are obtained by four-time repetition, respectively, and then the difference of these five zero points is taken as the difference between the latter and former terms to obtain $∆Z_{1}$, $∆Z_{2}$, $∆Z_{3}$, $∆Z_{4}$, as shown in Table 4.

The presence or absence of the weld cup affects the amount of strain in the strain region of the sensor and the hysteresis of the sensor. The hysteresis of the sensors with and without weld cup is listed in Table 5.

The special construction and mounting position of the elastomer and the weld cup makes it possible for the performance of the sensor to be affected by the weld cup. The elastomer and the weld cup look like a system of damping. The elastomer is like a spring, and the addition of the weld cup is like the addition of another spring. This affects the hysteresis of the entire sensor system, which in turn affects the return-to-zero error after the first load.

### 3.3. Elastomer Materials

The experimental results of Section 2.3.3 are shown in Table 6.

In the above Table 6, SS stands for steel sensors and AS stands for aluminum sensors.

The hysteresis and return-to-zero error after the first load are listed for all sensors, as shown in Table 7.

If the hysteresis is bigger, the return-to-zero error after the first load is also bigger. This is clearly shown in Table 7.

### 3.4. Analysis

The experimental results show that the variables are correlated and that the return-to-zero error after the first loading varies with the hysteresis, so the linear correlation between the two is analyzed. The degree of linear correlation between these two variables was measured using the Pearson correlation coefficient and the *p*-value [37]. The Pearson correlation coefficient was first designed as a statistical indicator by the statistician Carl Pearson to study the linear correlation between variables. Its value ranges from −1 to 1, with a value of 0 indicating that the two variables are not correlated, values greater than 0 and closer to 1 indicating a strong positive correlation between the two variables, and less than 0 and values closer to −1 indicating a strong negative correlation between the two variables. All the zero-return errors and hysteresis after the first loading from the experiment were analyzed to verify their correlation.

The hysteresis is x, the return-to-zero error after the first loading is y, and their sample datas are denoted as $x_{1}$, $x_{2}$, …, $x_{n}$ and $y_{1}$, $y_{2}$, …, $y_{n}$, where *n* is the number of samples. First, we need to compute the mean values $\bar{x}$ and $\bar{y}$ of x and y.

Finally, the formula for the Pearson correlation coefficient is cited to calculate r.

(1) $$r=\frac{Cov(x,y)}{\sigma x\cdot \sigma y}.$$

By calculation results, we obtain r=0.994, $P=6.122\times 10^{-9}$. It is clear that the return-to-zero error after the first loading and hysteresis belong to linear strong correlation.

Summary: Temperature has a small effect on the return-to-zero error after the first loading, which is due to the fact that the change in temperature causes a small change in the hysteresis. The sensors also do not have weld cup, is the elastomer material the same. All of this affects the hysteresis of the sensor system. Various factors affect the hysteresis first, and then the return-to-zero error after the first load of the load cell.

### 3.5. Fitting Hysteresis—First Load Return-to-Zero Error Curve

In order to make the effect between the hysteresis and the return-to-zero error after the first load clearer, we performed the same experiments as in Section 3 more times and obtained more realistic experimental data. After the data were obtained, the hysteresis of the sensor was set to be x, and the return-to-zero error after the first load was set to be y. A polynomial curve fitting algorithm was utilized to generate an approximation to the principal function of the x, y curve [38]. Polynomial curve fitting is often referred to as a data fitting algorithm or a data approximation algorithm. It is a widely used technique in mathematics and statistics for approximating a set of data points by a polynomial function. The experimental data are shown in Table 8.

**Figure 13:**
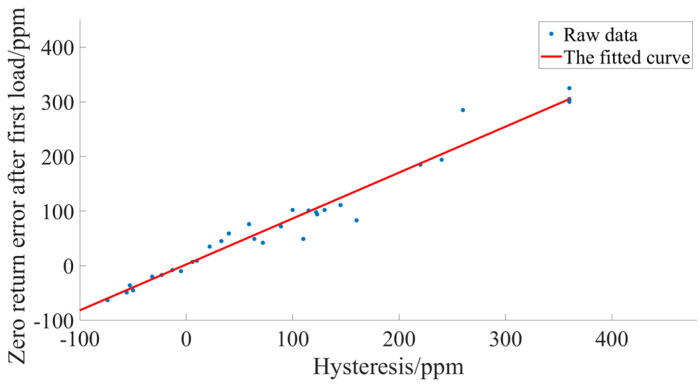
Curves of hysteresis with return-to-zero error after first loading.

The relationship plot after fitting the two sets of data by Polynomial Curve Fitting algorithm as shown in Figure 13.

The relationship equation obtained after fitting the two sets of data by the Polynomial Curve Fitting algorithm is as follows:

(2) y=0.8528x+2.245.

This also means that if the hysteresis is around −3 ppm, the return-to-zero error after the first loading is small.

## 4. Experimental Verification

In Section 3.4, it was determined that the return-to-zero error after the first loading is very small when the hysteresis is about −3 ppm. Therefore, we took some sensors with hysteresis of about −3 ppm for the experiment, and the experimental steps were the same as in Section 3.4. The experimental results are shown in Table 9.

**Figure 14:**
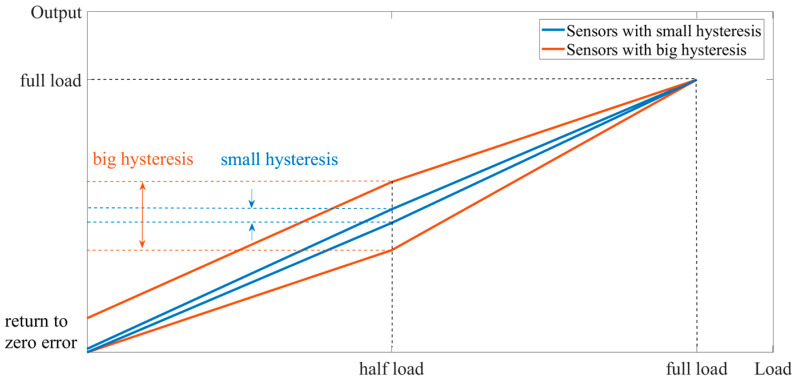
Comparison of sensor return to zero with large hysteresis vs. small hysteresis.

The data from the load and unload of the sensors used in the experiments were averaged, and then the curves were plotted and compared with the load and unload curves of the large hysteresis sensor, as shown in Figure 14.

Through experimental verification, the zero-return error after the first loading of the small hysteresis sensor was very small, which also verified the viewpoints proposed in this paper: the larger the hysteresis, the larger the zero-return error after the first loading. The smaller the hysteresis, the smaller the return-to-zero error after the first load.

## 5. Conclusions

In this paper, starting from the temperature, the material of the sensor, and whether the sensor has a weld cup or not, on the basis of the real experimental data, we introduce the relationship of the hysteresis to the influence of the return-to-zero error after the first loading and fit the relationship curve between the hysteresis and the return-to-zero error after the first loading on the basis of data processing. We summarize it as follows:

1. The influence of temperature on hysteresis is small, so the change in temperature has little influence on the return-to-zero error after the first loading.
2. The material characteristics are different and there is no weld cup. These factors affect the size of the hysteresis, which in turn affects the return-to-zero error after the first loading.
3. The Pearson correlation coefficient shows that the lag and the return-to-zero error after the first load are linearly and strongly correlated. The return-to-zero error after the first load are synthesized into a single function by means of a polynomial curve-fitting algorithm. It is proven that the smaller the hysteresis, the smaller the return-to-zero error after the first loading of the sensor.
4. This paper investigates the zero-point error of weight sensors and its relationship with hysteresis, which helps in compensating for the zero-point error. The purpose is to make the load cell more accurate when measuring the weight of an object.

In the manufacture of sensors, if the positive hysteresis of the elastomer and the negative hysteresis of the strain gauge can be canceled out so that the system hysteresis of the entire sensors are close to zero, the return-to-zero error after the first load will become very small. At the same time, the repeatability and accuracy of the load cell will be higher. 

## Figures and Tables

**Table 1:** Material Parameters.

Material Name	Density/(kg·m^−3^)	Young’s Modulus /GPa	Poisson’s Ratio
Q45	7890	200	0.269

**Table 2:** Zero point at different temperatures.

Temperature Conditions	Zero Point (g)
20 °C	0
After load at 20 °C	43
40 °C	−40
After load at 40 °C	7
−10 °C	35
After load at −10 °C	100
5 °C	−17
After load at 5 ℃	29
20 °C	−55
After load at 20 °C	−11

**Table 3:** Zero-point change after load and unload at the same temperature.

Temperature Conditions 9 (°C)	Δ*Z* (g)
20	43
40	47
−10	65
5	46
20	44

**Table 4:** Return-to-zero Error for With Weld cup Sensors and without Weld cup Sensors.

Zero Point Change	With Weld Cup	Without Weld Cup
Δ*Z*_1_ (ppm)	254.6	−54.6
Δ*Z*_2_ (ppm)	18.2	−18.2
Δ*Z*_3_ (ppm)	13.6	−9.1
Δ*Z*_4_ (ppm)	4.6	0

**Table 5:** Hysteresis for With Weld cup Sensors and without Weld cup Sensor.

Type	With Weld Cup	Without Weld Cup
Hysteresis (ppm)	305	−275

**Table 6:** Zero-point change in different sensors.

Number	SS1	SS2	SS3	SS4	SS5	AS1	AS2	AS3	AS4	AS5
Δ*Z*_1_ (ppm)	305	305	335	285	185	−63	−36	−71	−75	−20
Δ*Z*_2_ (ppm)	4	1	3	2	0	1	0	−1	−1	0
Δ*Z*_3_ (ppm)	1	0	0	1	0	0	0	0	0	0

**Table 7:** First return-to-zero error and hysteresis for two sensors.

Number	SS1	SS2	SS3	SS4	SS5	AS1	AS2	AS3	AS4	AS5
Δ*Z*_1_ (ppm)	305	305	335	285	185	−63	−36	−71	−75	−20
Hysteresis (ppm)	360	360	360	260	220	−74	−53	−104	−106	−32

**Table 8:** Relationship between hysteresis and return-to-zero error after first load.

X (ppm)	Y (ppm)	X (ppm)	Y (ppm)	X (ppm)	Y (ppm)	X (ppm)	Y (ppm)	X (ppm)	Y (ppm)
−106	−75	360	305	123	94	59	35	240	208
−104	−71	360	335	115	101	64	45	10	9
−74	−63	−13	−8	130	102	22	59	6	7
−53	−36	−23	−17	122	98	33	45	−5	−10
−32	−20	−50	−45	110	72	40	59		
220	285	−56	−49	72	42	160	153		
360	305	100	102	89	49	145	111		

**Table 9:** Return-to-zero error after first load for small hysteresis sensors.

Number	S1	S2	S3	S4	S5	S6
Hysteresis (ppm)	3	5	4	6	2	5
Δ*Z* (ppm)	2	1	4	2	0	2

## Data Availability

Not applicable.
